# Translationally Controlled Tumor Protein in Prostatic Adenocarcinoma: Correlation with Tumor Grading and Treatment-Related Changes

**DOI:** 10.1155/2015/985950

**Published:** 2015-01-15

**Authors:** Bruno Jim Rocca, Alessandro Ginori, Aurora Barone, Calogera Calandra, Filippo Crivelli, Giulia De Falco, Sara Gazaneo, Sergio Tripodi, Gabriele Cevenini, Maria Teresa del Vecchio, Maria Raffaella Ambrosio, Piero Tosi

**Affiliations:** ^1^Section of Pathology, Department of Medical Biotechnology, University of Siena, Via delle Scotte 6, 53100 Siena, Italy; ^2^Section of Pathology, Ospedale di Circolo di Busto Arsizio, Presidio Ospedaliero di Saronno, Piazzale Borella 1, 21047 Saronno, Italy; ^3^School of Biological and Chemical Sciences, Queen Mary University of London, Mile End Road, E14NS London, UK; ^4^Section of Pathology, Azienda Ospedaliera Universitaria Senese, Viale Bracci 16, 53100 Siena, Italy; ^5^Department of Medicine, Science and Neurosciences, University of Siena, Via delle Scotte 6, 53100 Siena, Italy

## Abstract

Prostate cancer is the second leading cause of cancer-related death. The androgen deprivation therapy is the standard treatment for advanced stages. Unfortunately, virtually all tumors become resistant to androgen withdrawal. The progression to castration-resistance is not fully understood, although a recent paper has suggested translationally controlled tumor protein to be implicated in the process. The present study was designed to investigate the role of this protein in prostate cancer, focusing on the correlation between its expression level with tumor differentiation and response to treatment. We retrieved 292 prostatic cancer specimens; of these 153 had been treated only by radical prostatectomy and 139 had undergone radical prostatectomy after neoadjuvant treatment with combined androgen blockade therapy. Non-neoplastic controls were represented by 102 prostatic peripheral zone specimens. In untreated patients, the expression of the protein, evaluated by RT-qPCR and immunohistochemistry, was significantly higher in tumor specimens than in non-neoplastic control, increasing as Gleason pattern and score progressed. In treated prostates, the staining was correlated with the response to treatment. An association between protein expression and the main clinicopathological factors involved in prostate cancer aggressiveness was identified. These findings suggest that the protein may be a promising prognostic factor and a target for therapy.

## 1. Introduction

Prostate cancer (PC) is the most commonly diagnosed male malignancy and the second leading cause of cancer-related death [1]. The disease course of PC is highly variable [2]. Most of the tumors develop slowly while others progress rapidly to life-threatening metastatic disease [3]. Organ confined PC is curable by surgery and radiation therapy and the prognosis for these patients is excellent [4]. The combined androgen blockade (CAB) is the standard treatment for inoperable patients with advanced-stage PC (regional lymph node involvement or metastasis) and for patients who underwent radiation therapy only [5, 6] since it inhibits androgen production in the testis with either chemical or surgical castration and blocks androgen receptor with an antiandrogen drug. It has been recently demonstrated that early adjuvant CAB delays the progression of clinical disease in patients with positive node on histology [7, 8]. Unfortunately, virtually all tumors become resistant to androgen withdrawal and develop into castrate-resistant PC (CRPC), which is associated with high morbidity and mortality [9]. The progression to CRPC is a process not fully understood [10]. It is well known that androgens are required for normal growth and functioning of the prostate gland by binding androgen receptors (AR) [11]. AR can modulate gene expression directly by interacting with specific elements in the regulatory regions of target genes or indirectly by activating various growth factor signaling pathways [12]. Most metastatic CRPC show mutations, amplifications, and deletions of the AR gene as well as conformational changes of the AR protein and sensitivity to growth factors and cytokines [13]. This leads to activation of AR without androgen [14]. An effective treatment for these patients is not yet available though a new drug (i.e., abiraterone acetate) seems to be promising [15]. Additional therapeutic strategies targeting molecular mechanisms-mediating resistance are required, either to delay or to prevent the emergence of the castration resistant phenotype [16]. The translationally controlled tumor protein (TCTP) is a protein highly expressed in mammals and in a wide range of other organisms. The conservation of TCTP-converging network through phylogeny underscores its relevance [17]. All the processes regulated by the protein, including promoting cell growth, activating components of the mTOR pathway, inhibiting BOX homodimerization and apoptosis, antagonizing p53, and being overexpressed in tumors in respect to the normal counterpart, may converge to a limited set of key events that control stemness pluripotency, tumor reversion, cell fate determination, and ultimately tumorigenesis [17]. Therefore, TCTP combines both a tumor suppressive and an oncogenic activity that results in a context-dependent cancer phenotype, representing a check point and a switch necessary for cellular reprogramming [17]. We have previously demonstrated the presence of both TCTP mRNA and protein in prostatic tissue, in prostate cancer cell lines and in the prostatic fluid [18], suggesting specific roles for the protein such as apoptosis and control of sperm functions. Recent studies have proposed TCTP as an androgen-regulated gene implicated in PC, providing preclinical proof-of-principle that combining antisense oligonucleotide-mediated TCTP knockdown with castration and/or docetaxel therapy could serve as a novel strategy to treat CRPC [9, 19]. However, it is not currently known whether there is a correlation between the different histological grades of PC and TCTP expression or whether the protein is expressed in preinvasive lesions as high-grade prostatic intraepithelial neoplasia (HGPIN). Therefore, the present study was designed to investigate TCTP expression in prostate carcinoma, focusing on the correlation between protein/gene expression level and tumor differentiation. Furthermore, TCTP expression was also studied in neoplastic tissue after androgen deprivation to obtain more information on the hormonal regulation of this protein.

## 2. Materials and Methods

### 2.1. Patients

Ethics approval for this study was obtained from the Institutional Review Board at the University of Siena (Italy). Informed written consent was gained from the patients and all specimens were handled and made anonymous. We retrieved from the archives of Siena University Hospital (Siena, Italy) prostate needle biopsies and the corresponding radical prostatectomy of 292 patients who had undergone surgery for PC between January 1999 and December 2003. Of these, 153 patients had been treated only by radical prostatectomy and 139 had undergone radical prostatectomy after neoadjuvant treatment with androgen-deprivation therapy. Non-neoplastic controls were represented by 102 prostatic peripheral zone specimens of patients who underwent cystoprostatectomy for bladder cancer but with no tumor in prostate gland. The mean age of the patients at the time of surgery was 69 years (range: 55 to 79 years). The following biochemical and pathological parameters were also recorded: total prostatic specific antigen (tPSA), Gleason score (in both needle biopsies and surgical specimens for untreated patients, and only in needle biopsies for treated patients), surgical margins infiltration, extraprostatic extension, seminal vesicles invasion, lymph node metastasis, and TNM staging system (based on the AJCC Cancer Staging Manual, Seventh Edition, 2010, Springer New York, Inc.). The clinicopathological features of the 292 patients are summarized in Table 1.

### 2.2. Histology

Core needle biopsies and surgical specimens had been fixed in 10% buffered formalin and embedded in paraffin. From each block, 4 *μ*m thick sections had been cut and stained with haematoxylin and eosin (H&E). All the slides were reviewed by three expert uropathologists (MTdV, FC, and CC), who subsequently met to obtain a consensus diagnosis. Tumor grading was established according to the updated Gleason grading system in each sample [20, 21]. Representative tumor sections were then classified as low grade when the combined Gleason score (primary plus secondary pattern) was ≤7 and as high grade when the combined Gleason score was ≥8. Foci of HGPIN were also identified, when present in peritumoral areas. Treated adenocarcinoma patients were classified as good, moderate, or poor responders on the basis of the changes in the morphological parameters suggested by Montironi et al. (nuclear enlargement, prominent nucleoli, cell cytoplasm vacuolization, acinar shrinkage, isolated infiltrating tumor cells, difficulty in recognizing prostate cancer patterns, and amount of interstitial tissue stroma) [22].

### 2.3. RT-qPCR for TCTP Expression

The expression of TCTP was evaluated in non-neoplastic and neoplastic prostate cancers. In particular, neoplastic samples were representative of different Gleason pattern (Gleason patterns 3–5) and score and of different response to treatment (poor, moderate, and good responder patients). Tumor cells corresponding to different Gleason pattern were isolated by laser capture microdissection on H&E-stained sections (4-5 *μ*m thick) from formalin-fixed paraffin-embedded tissues, using a laser capture microdissector (Arcturus, MWG, Florence, Italy). Microdissected cells were transferred to a Capsure transfer film, containing 200 *μ*L of Trizol (Invitrogen, CA). RNA was then extracted following manufacturer's instructions. Reverse transcription was carried out using the Quantitect Reverse transcription Kit (Qiagen, CA). For each RNA specimen, a negative control was prepared by omitting the reverse transcriptase. TCTP expression was analyzed both in non-neoplastic and neoplastic samples by RT-qPCR, using Taqman probes (Life Sciences, Applied Biosystems, CA) for TCTP and HPRT as housekeeping gene, according to the manufacturer's instructions. Relative quantification was calculated by the ΔΔCt method [23].

### 2.4. Immunohistochemistry

The most representative tumor blocks were selected on the basis of the morphological features, and the highest Gleason pattern and score were chosen both in needle biopsies and in radical prostatectomy for each nontreated patient. Since Gleason score is not applicable in treated patients because CAB determines a pronounced simplification of architectural pattern [22], each specimen was selected depending on the response to treatment (poor, moderate, and good responder patients). Immunohistochemical stainings were performed on 4 ± 0.5 *μ*m thick sections of each block employing the Ultravision Detection System Antipolyvalent HRP (Ultra V Block) (LabVision, Fremont, CA, USA, Bio-Optica). All the procedures were carried out automatically by using the Bond-III machine. Slides were incubated with an anti-TCTP antibody (dilution: 1 : 25) and the reaction revealed using fucsin (Dako, Milan, Italy) as chromogen. Sections were weakly counterstained with Harris' haematoxylin and examined under a light microscope. Nonimmune serum immunoglobulins were used as negative control, whereas the positive control was represented by placental tissue [24].

### 2.5. Staining Assessment

All of the samples were independently evaluated and scored by two investigators (MRA and BJR), who were blinded to the clinicopathological information of the patients. TCTP protein expression levels were classified semiquantitatively combining the proportion and intensity of positively stained cells [25–27]. The percentage of positive-staining tumor cells was scored as follows: 1 (<5% positive cells), 2 (5–50% positive cell), and 3 (>50% positive cells) [25–27]. Staining intensity was scored as follows: 1 (weak or not detectable staining), 2 (moderate staining), and 3 (strong staining) [25–27]. Three different fields (at least 100 cells/field) were evaluated at ×200 magnification. In PC samples TCTP protein expression level was evaluated and defined only in neoplastic cells. The sum of the staining intensity score and the percentage score was used to define the TCTP protein expression level, low: 0–2; high: 3-4. The agreement between the two pathologist was about 90%. Cases with discrepancies were reviewed and discussed to reach the 100% of concordance. In core needle biopsies and in untreated radical prostatectomies, staining assessment was performed separately in the two patterns (i.e., 3 and 4 in Gleason score 7, and 4 and 5 in Gleason score 9). In treated radical prostatectomies, TCTP expression was evaluated in comparison to the extent of histological response to treatment.

### 2.6. Statistical Analysis

Descriptive statistics was computed, including frequency count, minimum, maximum, mean, and standard deviation for quantitative variables and frequency count and percentage for qualitative variables.

The Kolmogorov-Smirnov test was applied to verify normal distribution of quantitative variables age and PSA. When normality was assessed, one-way analysis of variance (ANOVA) was used to compare the two TCTP levels, with post hoc test of Bonferroni for pairwise comparisons. For nonnormal data we otherwise used the nonparametric tests of Kruskal-Wallis and the Dunn post hoc test.

The Kendall rank correlation coefficient, *τ*, was computed to evaluate the correlation between TCTP levels and all the at-least-ordinal variables.

When computable, the Fisher exact test was applied to contingency tables to evaluate the association between the frequency distributions of TCTP levels and each qualitative variable. We alternatively used the classic *χ* ^2^ test.

The prognostic power of TCTP, with relation to the disease-free survival (DFS) time, was investigated by using the Cox proportional hazards model. The multivariate model was designed in a stepwise manner, by including at any successive step only the prognostic factors (covariates) that could be associated with survival with a statistical significance greater than 95% (*P* < 0.05). The stepwise method allows univariate survival analysis to be also evaluated at step 0. The hazard ratio (HR) and its 95% confidence interval were computed for each prognostic factor, for both univariate and multivariate analyses. HR represents the odds that an individual in the group with the highest risk reaches the endpoint first. Finally, the Kaplan-Meier DFS curves for the two TCTP levels were drawn and statistically compared through the log rank test.

In each group, the difference in TCTP expression between needle biopsy and radical prostatectomy was evaluated.

Results were considered statistically significant when they exceeded the 95% probability level (*P* < 0.05).

## 3. Results

### 3.1. TCTP mRNA in Untreated and Treated PC

**Figure 1:**
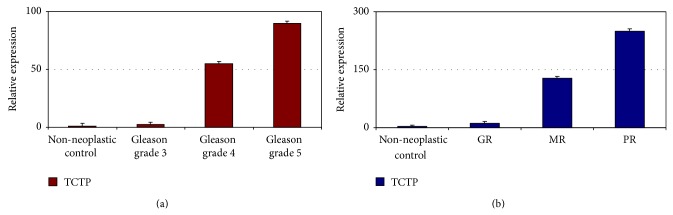
Relative expression of TCTP by RT-qPCR in tumors of different pattern in respect to normal control. In untreated patients, higher expression is observed in cancer, in respect to non-neoplastic control, with an increase as tumor grade progresses (a) (*t*-test, *P* < 0.05). In treated patients, we found that the more the treatment is effective, the less the expression level of TCTP is, which is almost comparable to non-neoplastic control in GR (b) (GR: good responders; MR: moderate responders; PR: poor responders) (*t*-test, *P* < 0.05). The graphs are representative of three different experiments. Error bars indicate standard deviation.

The expression of TCTP in non-neoplastic and neoplastic prostate samples was evaluated by RT-qPCR. Relative quantification indicated that the expression of TCTP is significantly higher in tumor specimens than in non-neoplastic control, increasing as Gleason pattern (Figure 1(a)) and Gleason score progressed (*t*-test, *P* < 0.05). In patients treated with CAB, TCTP expression is correlated to response to therapy, being higher in poor responders and lower in good responders (Figure 1(b)) (*t*-test, *P* < 0.05).

### 3.2. TCTP Protein Expression in Untreated and Treated PC

**Figure 2:**
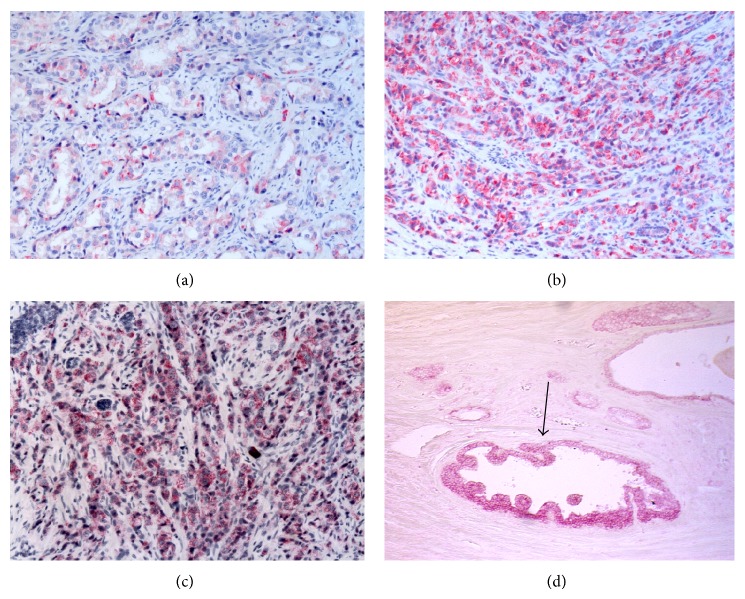
TCTP protein expression in untreated patients. TCTP staining increases as Gleason pattern progresses from Gleason pattern 3 (a) to Gleason pattern 4 (b) to 5 (c); in the latter isolated neoplastic cells strongly express the protein (*χ* ^2^-test, *P* < 0.001). High positivity is also present in associated-cancer HGPIN ((d), arrow) in respect to the non-neoplastic tissue. (Original Magnification, O.M.: (a)–(d), 10x).

**Figure 3:**
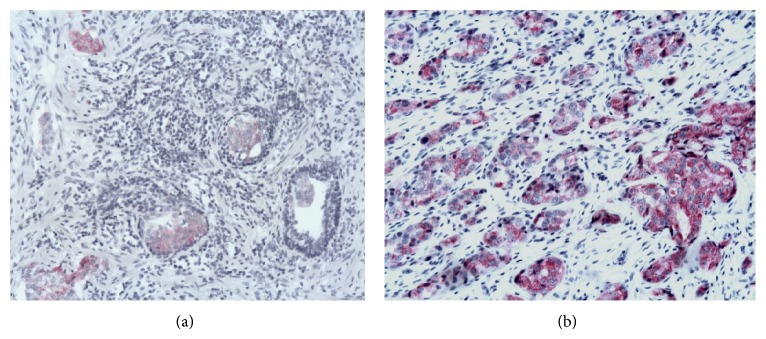
TCTP protein expression in treated patients. The intensity of immunostaining is absent or low in good responders (a) and high in poor responders (b) (Kendall's *τ*: 0.773, *P* < 0.001). (O.M., (a)-(b), 10x).

Among the non-neoplastic specimens, 78 showed atrophy and 24 atrophy plus chronic inflammation. A low TCTP expression was detected in the cytoplasm of the epithelial cells, mainly located in the apical portion and blebs, whereas the basal layer cells showed a strong positivity. Among the 153 untreated PC, 57 were Gleason score 6, 52 Gleason score 7, 25 Gleason score 8, 17 Gleason score 9, and 2 Gleason score 10. For the Gleason scores 7 and 9, TCTP protein expression was also evaluated separately in the two patterns (i.e., 3 and 4 for Gleason score 7 and 4 and 5 for Gleason score 9). A correlation between TCTP immunostaining and Gleason pattern and score was observed. TCTP protein expression was higher in high Gleason pattern (i.e., 4 and 5) in comparison to low Gleason pattern (i.e., 3) (*P* < 0.04) (Figures 2(a)–2(c)). As far as Gleason score is concerned, TCTP immunostaining was significantly lower in Gleason score 6 PC (*P* < 0.001) and stronger in Gleason score 8 to 10 PC (*P* < 0.001) (Table 2(a)). In Gleason score 7 PC an intratumoral heterogeneity was observed, with a higher TCTP protein expression in Gleason pattern 4 than in Gleason pattern 3. The stromal cells both in non-neoplastic and in neoplastic specimens showed a mild staining. Strong staining was observed in cancer-associated HGPIN (Figure 2(d)). No differences in TCTP protein expression were observed between the needle biopsies and the corresponding radical prostatectomy of the same Gleason pattern. In treated prostates, 48 were good responders, 40 were moderate responders, and 51 were poor responders. The intensity of TCTP immunostaining was higher in poor responders and lower in good responders (Table 2(b) and Figures 3(a)-3(b)) (Kendall's *τ*: 0.773, *P* < 0.001).

### 3.3. Association of TCTP Protein Expression with the Prognostic Factors of Prostatic Carcinoma

**Figure 4:**
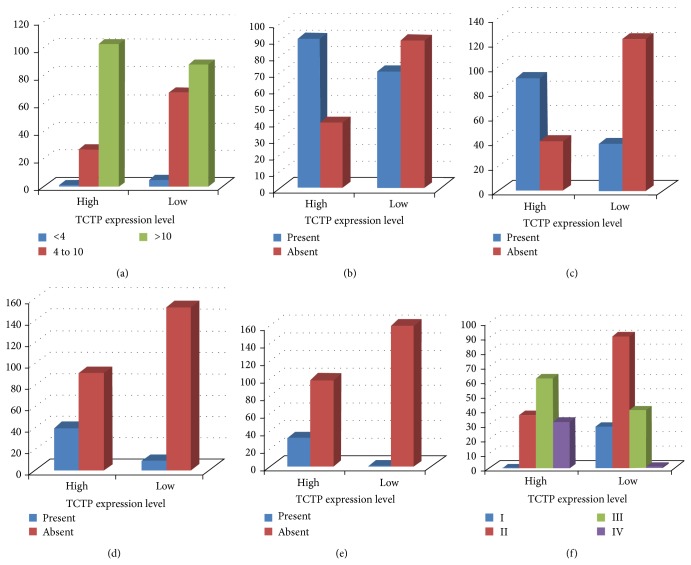
Association of TCTP protein expression with the main prognostic factors involved in PC aggressiveness. Univariate analysis by Fisher exact test and/or *χ* ^2^-test shows that TCTP protein expression level is significantly associated with higher preoperative PSA (a) (*P* < 0.05), surgical margins infiltration (b) (*P* < 0.001), extraprostatic extension (c) (*P* < 0.001), seminal vesicles invasion (d) (*P* < 0.001), lymph node metastasis (*P* < 0.001) (e), and higher TNM stage (f).

We investigated the association between TCTP protein expression status and the well-recognized prognostic factors of PC. The intensity of TCTP was directly correlated with higher preoperative PSA (*P* < 0.05), stage (*P* < 0.001) and Gleason score (*P* < 0.001), surgical margins infiltration (*P* < 0.001), extraprostatic extension (*P* < 0.001), seminal vesicles invasion (*P* < 0.001), and lymph node metastasis (*P* < 0.001). On the contrary, no correlation has been identified between TCTP expression and the age of the patients (*P* = 0.99) (Figures 4(a)–4(f)).

### 3.4. Disease-Free Survival Analysis

**Figure 5:**
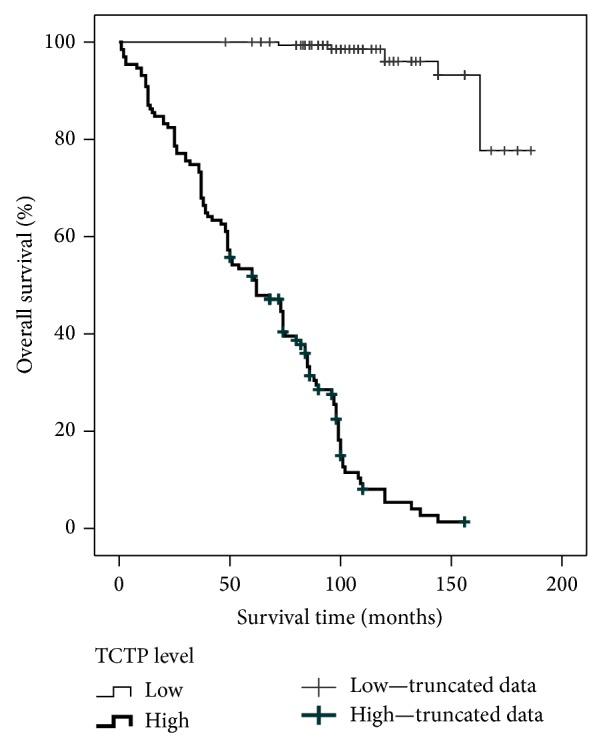
Kaplan-Meier curves of TCTP protein expression levels. Disease free survival was significantly different between the groups with high and low TCTP protein expression (log rank test, *P* < 0.001). The group with the higher TCTP protein expression has a shorter disease free survival (mean = 64 months) when compared to the group with the lower TCTP protein expression (mean = 178 months).

The Kaplan-Meier curves of TCPC protein expression are shown in Figure 5. DFS was significantly different between the groups with high and low TCTP expression (log rank test, *P* < 0.001). In particular, the group with the higher TCTP protein expression had a shorter DFS (mean = 64 months; 95% CI = 57–70 months) when compared to the group showing the lower TCTP protein expression (mean = 178 months; 95% CI = 171–185 months). Univariate Cox analysis showed that all the prognostic factors known to be involved in PC are highly statistically correlated with DFS (*P* < 0.001) except for the age (*P* = 0.73). In addition, we found no differences in DFS between the treated and the untreated patients (*P* = 0.32), thus confirming that the neoadjuvant treatment does not affect patients' prognosis. Stepwise multivariate analysis enrolling the above mentioned parameters and the TCTP protein staining demonstrated that TCTP was not an independent prognosticator but influences patients' outcome (HR = 49.7, CI 95% = 19.4–127.1 for high TCTP staining).

## 4. Discussion

TCTP is a multifaceted protein which has been implicated in a number of key cellular processes, both physiologic and pathologic, such as development, immune response, cell cycle, cell proliferation and growth, cell division, cytoskeleton activity, protein synthesis, and calcium binding [28, 29]. Recent studies have shown the contribution of TCTP in cell growth and proliferation of prostate cancer, suggesting TCTP as a novel possible therapeutic target in the treatment of castration-resistant PC [9, 19]. We have previously demonstrated TCTP expression in the human prostate cancer cell lines, LNCaP and PC-3, and in the non-neoplastic human prostate epithelial cells, PwR-1E [18] as well as in prostatic tissues from patient who underwent adenomectomy for benign prostatic hyperplasia. In the present study we have confirmed our previous data on a larger series of primary tumors by RT-qPCR and immunohistochemistry.

To the best of our knowledge, no previous papers have analyzed the correlation between TCTP expression and tumor grading as well as the influence that CAB may exert on the expression of this protein in PC. In our study we show that in HGPIN adjacent to adenocarcinoma TCTP expression was always strong, both in treated and untreated prostate. The overexpression of this protein in the initial phases of neoplastic transformation may support the hypothesis that TCTP could exert a role in tumorigenesis. In untreated prostate, the most striking evidence is that TCTP expression significantly correlates with tumor grading, being high in Gleason score ≥8 PC and low in Gleason score ≤7 PC, indicating a possible correlation with cell differentiation. Interestingly, in Gleason score 7, TCTP expression shows areas of different distribution and intensity, being stronger in Gleason pattern 4 than in Gleason pattern 3. This underlines the heterogeneity of PC and may explain why Gleason score 7 prostatic carcinomas with the same stage, but with different extension of Gleason pattern 4, may have different clinical behaviors. One should speculate that the aggressiveness of the tumor may really depend on this parameter. In untreated patients we do not identify differences in the expression of TCTP between needle biopsies and the corresponding radical prostatectomy, in cases with the same Gleason pattern and score; therefore TCTP may represent a promising tool to be used in initial needle biopsies to predict the biological aggressiveness of the tumors and to select an optimal therapeutic treatment for each patient [19]. In treated patients we find a correlation between the intensity and expression of TCTP in cancer and the response to CAB. This may explain the initial good effect of hormonal treatment and may confirm previous study demonstrating the utility of early adjuvant CAB in advanced disease [7]. However, we do not know whether good response to treatment may be related to TCTP's androgen regulation or to sensitivity to androgen withdrawal and if other androgen related genes may behave in a similar manner.

Univariate analysis shows that Gleason score, TNM stage, and TCTP expression have a statistically significant correlation with DFS. Among these parameters, multivariate analysis identifies Gleason score and TCTP protein expression as the best predictors, when combined, of aggressiveness of PC.

## 5. Conclusions

Collectively, our data demonstrate that, in PC, TCTP expression is directly correlated with tumor differentiation and with the main clinicopathological factors involved in PC aggressiveness. The assessment of TCTP staining in needle biopsies from PC patients may be of help in evaluating the more appropriate therapeutic strategies.

## Figures and Tables

**Table 1:** Main characteristics of the patients included in this study. Univariate descriptive statistics (including frequencies and percentages) of each qualitative clinical parameter in relation to the two TCTP protein expression levels is shown with the relative *P* value. A statistically significant association between TCTP protein expression and the main prognostic factors involved in PC aggressiveness is detected.

Variable	Group	*N*	TCTP protein expression		*P* value
			High	Low	
Preoperative PSA	<4	6 (2%)	1 (0.8%)	5 (3.1%)	<0.05
	4–10	95 (32.5%)	27 (20.6%)	68 (42.2%)	
	>10	191 (65.5%)	103 (78.6%)	88 (54.7%)	

Surgical margins infiltration	Present	162 (55.5%)	91 (69.5%)	71 (44.1%)	<0.001
	Absent	130 (44.5%)	40 (30.5%)	90 (55.9%)	

Extraprostatic extension	Present	129 (44.2%)	91 (69.5%)	38 (23.6%)	<0.001
	Absent	163 (55.8%)	40 (30.5%)	123 (76.4%)	

Seminal vesicles invasion	Present	49 (16.8%)	40 (30.5%)	9 (5.6%)	<0.001
	Absent	243 (83.2%)	91 (69.5%)	152 (94.4%)	

Lymph node metastasis	Present	34 (11.6%)	33 (25.2%)	1 (0.6%)	<0.001
	Absent	258 (88.4%)	98 (74.8%)	160 (99.4%)	

TNM stage	I	29 (9.9%)	0 (0%)	29 (18%)	<0.001
	II	128 (43.9%)	37 (28.2%)	91 (56.5%)	
	III	102 (34.9%)	62 (47.4%)	40 (24.9%)	
	IV	33 (11.3%)	32 (24.4%)	1 (0.6%)	

Recurrence	Present	120 (41.4%)	114 (87%)	6 (3.7%)	<0.001
	Absent	172 (58.6%)	17 (13%)	155 (96.3%)	

PC: prostatic cancer; *N*: number of cases and percentage.

**(a):** 

Gleason score	*N*	%	TCTP protein expression	
			High	Low
6	57	37.3	4 (7%)	53 (93%)
7	52	34	28 (53.8%)	24 (46.1%)
8	25	16.3	24 (96%)	1 (4%)
9	17	11.1	17 (100%)	0 (0%)
10	2	1.3	2 (100%)	0 (0%)

**(b):** 

Response to treatment	*N*	%	TCTP protein expression	
			High	Low
Poor	51	36.7	50 (98%)	1 (2%)
Moderate	40	28.8	24 (60%)	16 (40%)
Good	48	34.5	2 (4.2%)	46 (95.8%)
